# InspeCKD – Analyse zur Prävalenz, Diagnose und Therapie der chronischen Nierenerkrankung: Daten von Risikopatienten in deutschen Hausarztpraxen

**DOI:** 10.1007/s00108-025-01958-6

**Published:** 2025-08-04

**Authors:** Christoph Wanner, Elke Schaeffner, Thomas Frese, Ulrich Opfermann, Frank Radowsky, Philipp Stahl, Fabian Burckhardt, Felix Scherg, Christoph Weber, Frederik Mader

**Affiliations:** 1 Abteilung klinische Studien und Epidemiologie, Deutsches Zentrum für Herzinsuffizienz, Universitätsklinikum Würzburg, Am Schwarzenberg 15, 97078 Würzburg, Deutschland; 2 Institut für Public Health, Charité – Universitätsmedizin Berlin, Berlin, Deutschland; 3 Institut für Allgemeinmedizin, Universitätsklinikum Halle (Saale), Halle, Deutschland; 4 SYMEDICUM MVZ GmbH, Berlin, Deutschland; 5 Allgemeinmedizinische Praxis Radowsky, Leipzig, Deutschland; 6 Hausärztliche Gemeinschaftspraxis Meinecke & Stahl, Burg, Deutschland; 7 BioPharmaceuticals Medical, AstraZeneca, Hamburg, Deutschland; 8 Praxis Rheinlanddamm, Dortmund, Deutschland; 9 Gemeinschaftspraxis Nittendorf, Nittendorf, Deutschland; 10 IU Internationale Hochschule, Erfurt, Deutschland

**Keywords:** Chronische Nierenerkrankung/Risikofaktoren, Chronische Nierenerkrankung/Früherkennung, Primärversorgung, Renin-Angiotensin-System-Hemmer, Natrium-Glukose-Kotransporter-2-Hemmer, Chronic kidney disease/risk factors, Chronic kidney disease/early diagnosis, Primary health care, Renin-angiotensin system inhibitors, Sodium-glucose transporter 2 inhibitors

## Abstract

**Hintergrund:**

Patienten mit Bluthochdruck, Diabetes mellitus (DM) und/oder kardiovaskulären Erkrankungen („cardiovascular diseases“ [CVD]) sind besonders gefährdet, eine chronische Nierenerkrankung („chronic kidney disease“ [CKD]) zu entwickeln, und sollten daher regelmäßig auf eine CKD untersucht werden. Eine frühzeitige Diagnose und Behandlung der CKD kann das Risiko für Nierenversagen und kardiorenale Komplikationen senken.

**Ziel der Arbeit:**

Ziel der Querschnittsstudie war es, ein besseres Verständnis über die Prävalenz, Diagnostik, Diagnose und Therapie der CKD bei Risikopatienten in deutschen Hausarztpraxen zu gewinnen.

**Material und Methoden:**

Von 1244 Hausärzten wurden elektronische, vollständig anonymisierte Einzeldatensätze für die Analyse zur Verfügung gestellt (Studienzeitraum: 6/2020–6/2023). Eingeschlossen wurden gemäß den Screeningempfehlungen von Kidney Disease: Improving Global Outcomes (KDIGO) CKD-Risikopatienten mit Bluthochdruck und/oder DM und/oder CVD mit einer Beobachtungsdauer von mindestens einem Jahr.

**Ergebnisse:**

Die CKD-Prävalenz betrug 18,8 % (*n* = 24.179), wobei 16,5 % (*n* = 3986) eine Diagnose für CKD gemäß Internationaler statistischer Klassifikation der Krankheiten und verwandter Gesundheitsprobleme (ICD-10) hatten. Somit blieben 83,5 % (*n* = 20.193) der Risikopatienten ohne ICD-10-CKD-Diagnose. Bis 6 Monate nach Diagnosestellung wurden 9,7 % (*n* = 1740) der nach ICD-10 diagnostizierten CKD-Patienten mit einem Renin-Angiotensin-System-Hemmer in Kombination mit einem Natrium-Glukose-Kotransporter-2-Hemmer behandelt.

**Schlussfolgerung:**

Die Ergebnisse verdeutlichen Defizite in der Früherkennung und Therapie der CKD in Deutschland. Eine stärkere Sensibilisierung der Hausärzte für dieses unterschätzte Krankheitsbild ist dringend erforderlich.

**Graphic abstract:**

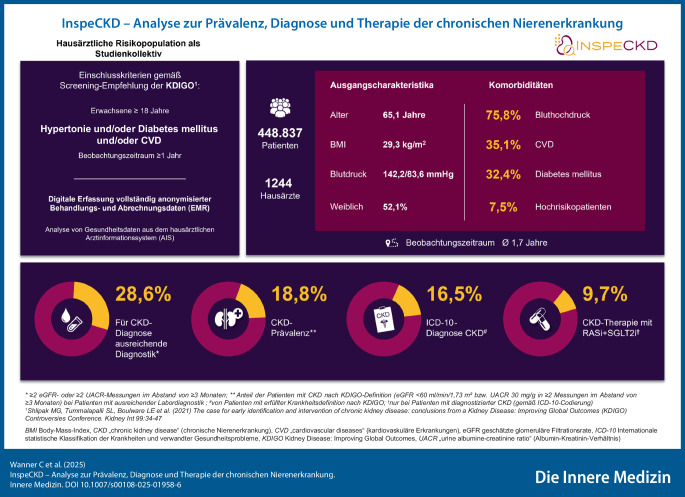

## Abstract (en)

**Background:**

Patients with hypertension, diabetes mellitus (DM) and/or cardiovascular disease (CVD) have a higher risk of developing chronic kidney disease (CKD) and should therefore be closely monitored. Early diagnosis and guideline-directed disease management can reduce the risk of kidney failure and cardiorenal complications.

**Objectives:**

The aim of this retrospective cross-sectional study was to gain a better understanding of the prevalence, diagnosis, and guideline-directed treatment of CKD in patients at risk for CKD in German general practitioner (GP) practices.

**Materials and methods:**

A total of 1244 GPs provided fully anonymized electronic data sets for the analysis (study period 06/2020–06/2023). According to the screening recommendations of Kidney Disease: Improving Global Outcomes (KDIGO), adults with hypertension and/or DM and/or CVD with a documented observation period of at least 1 year were included in the study.

**Results:**

The prevalence of CKD was 18.8% (*n* = 24,179), of which 16.5% of at-risk patients (*n* = 3986) had a documented diagnosis of CKD according to the International Statistical Classification of Diseases and Related Health Problems (ICD-10). Thus, 83.5% (*n* = 20.193) of patients at risk remained undiagnosed. Within 6 months of diagnosis, 9.7% (*n* = 1740) of ICD-10-diagnosed CKD patients were treated with a renin-angiotensin system inhibitor (RASi) in combination with a sodium-glucose cotransporter‑2 inhibitor (SGLT2i).

**Conclusion:**

The results highlight substantial gaps in early diagnosis and treatment of CKD in Germany. There is an urgent need for greater awareness among GPs of this under-recognized disease.

Weltweit leben etwa 850 Mio. Menschen mit einer chronischen Nierenerkrankung („chronic kidney disease“ [CKD]; [1]). In Deutschland wird die Prävalenz der CKD auf 12,7 % geschätzt, was, ohne Berücksichtigung des jeweiligen Krankheitsstadiums, etwa 10 Mio. Betroffenen entspricht [2]. Die steigende Prävalenz ist einerseits auf den demografischen Wandel zurückzuführen, andererseits nimmt die Zahl der Patienten mit Risikofaktoren für eine CKD, beispielsweise mit Bluthochdruck, Diabetes mellitus (DM) oder kardiovaskulären Erkrankungen („cardiovascular diseases“ [CVD]), zu [3, 4]. Die wenigsten Risikopatienten sind sich ihrer Erkrankung jedoch bewusst: Nur jeder dritte Patient weiß von seiner Nierenfunktionseinschränkung, und nur zwei von drei Patienten mit diagnostizierter CKD. Der Anteil an Patienten mit CKD im Stadium 3, die eine nach Internationaler statistischer Klassifikation der Krankheiten und verwandter Gesundheitsprobleme (ICD-10) codierte Diagnose erhalten, wird in Deutschland mit 16–47 % angegeben [5–8].

Nur jeder dritte Patient weiß von seiner Nierenfunktionseinschränkung

Da die CKD klinisch lange Zeit symptomlos verläuft, wird sie in den frühen Stadien selten aufgrund von Beschwerden diagnostiziert und bleibt daher ohne Labordiagnostik mit Blut- und Urinuntersuchungen oft unentdeckt [9]. Zwei Parameter sind dabei von besonderer Bedeutung [10]:

- Geschätzte glomeruläre Filtrationsrate (eGFR), berechnet aus der Kreatininkonzentration im Blutserum
- Albuminausscheidung im Spontanurin, gemessen als Albumin-Kreatinin-Verhältnis („urine albumine-creatinine ratio“ [UACR])

Obwohl die eGFR eine Abschätzung der Filtrationsleistung der Niere erlaubt, kann die Nierenfunktion bei einer bestehenden Nierenschädigung durch Kompensationsmechanismen über einen längeren Zeitraum aufrechterhalten werden. Eine Nierenschädigung macht sich daher häufig erst im fortgeschrittenen Stadium durch einen starken Abfall der eGFR bemerkbar [11]. Dagegen ist eine erhöhte Albuminausscheidung, gemessen anhand der UACR, ein frühzeitiger und eGFR-unabhängiger Hinweis auf eine Nierenschädigung [12]. Die Nationalen VersorgungsLeitlinien zu Diabetes mellitus Typ 2 und Hypertonie sowie die aktualisierte S3-Leitlinie „Versorgung von Patient*innen mit chronischer, nicht-nierenersatztherapiepflichtiger Nierenkrankheit in der Hausarztpraxis“ der Deutschen Gesellschaft für Allgemeinmedizin und Familienmedizin (DEGAM) empfehlen bei Risikopatienten ein CKD-Screening anhand von eGFR und UACR [13–15]. Die Ergebnisse der ersten Teilanalyse der InspeCKD-Studie zeigen eine Unterdiagnostik und somit Defizite beim CKD-Screening von Risikopatienten auf. Im Beobachtungszeitraum von 1,7 Jahren wurde bei weniger als der Hälfte (45,5 %) der Risikopatienten mindestens eine Serumkreatininmessung zur Berechnung der eGFR durchgeführt. Die UACR als wichtiger Früherkennungsmarker für eine CKD wurde bei 0,4 % der Risikopatienten bestimmt [16]. Dies lässt vermuten, dass die Dunkelziffer von Patienten mit unentdeckter CKD möglicherweise höher ist als bisher angenommen.

Eine erhöhte Albuminausscheidung ist ein früher, eGFR-unabhängiger Hinweis auf eine Nierenschädigung

Wird die CKD nicht frühzeitig diagnostiziert und behandelt, schreitet sie voran und bringt schwerwiegende gesundheitliche Komplikationen wie Dialysepflichtigkeit mit sich. Da die CKD auch ein bedeutender kardiovaskulärer Risikofaktor ist, haben Patienten mit CKD – je nach Krankheitsstadium – ein erhöhtes Risiko für Herz-Kreislauf-Erkrankungen und damit verbundene kardiorenale Komplikationen [17, 18]. Bei frühzeitiger Diagnose stehen mit Hemmstoffen des Renin-Angiotensin-Systems (RASi) und des Natrium-Glukose-Kotransporters 2 (SGLT-2i) wirksame Therapeutika zur Verfügung, mit denen die Gesamtmortalität und das Risiko für kardiorenale Komplikationen gesenkt, das Fortschreiten der Erkrankung bis hin zum Nierenversagen verlangsamt und die Lebensqualität verbessert werden können [19, 20].

CKD-Risikopatienten mit arterieller Hypertonie, DM und kardiovaskulären Vorerkrankungen sind Patienten, die in der täglichen Routine von Hausärzten behandelt werden. Eine frühzeitige Erkennung und die Initiierung einer medikamentösen CKD-Therapie bei diesen Patienten ist von entscheidender Bedeutung.

## Fragestellung und Zielsetzung

Ziel der InspeCKD-Studie war es, die Versorgungssituation (einschließlich diagnostischer Verfahren) von Patienten mit erhöhtem CKD-Risiko in der hausärztlichen Routineversorgung in Deutschland zu untersuchen. Gegenstand dieser hier vorliegenden Teilanalyse war es, den Anteil der Patienten, die bei Vorliegen einer CKD auch eine entsprechende ICD-10-Diagnose erhalten, zu analysieren. Zusätzlich sollten diese ICD-10-Prävalenzen und die sich anschließende medikamentöse CKD-Therapie in präspezifizierten (Sub‑)Populationen verglichen werden.

## Methode

### Studiendesign

InspeCKD (Secondary Data Analysis of Patients at Risk for CKD to Inspect CKD Prevalence, Diagnosis Rates, Diagnostic Behaviour, Treatment Patterns and Patient Characteristics) ist eine Querschnittsstudie zur Analyse vollständig anonymisierter Behandlungs- und Abrechnungsdaten (unter anderem demografische Daten, Laborparameter, Diagnosen, Medikation und Verordnungen), die bei ärztlichen Routinebesuchen im Arztinformationssystem (elektronische Patientenakte [EMR]) dokumentiert wurden (Studienzeitraum 6/2021–6/2023). Die erhobenen Daten wurden bereits lokal vor der Datenübermittlung im Arztinformationssystem der Praxis anonymisiert. Ein Rückschluss auf einen konkreten Patienten oder den behandelnden Hausarzt ist somit nicht möglich. Für die Analyse wurden lediglich Daten berücksichtigt, die ab dem Datum der ersten EMR-Übertragung in die Studiendatenbank maximal 24 Monate zurückreichten.

An der Studie nahmen insgesamt 1244 Hausärzte und hausärztlich tätige Internisten teil, die hinsichtlich der Region der Kassenärztlichen Vereinigung (KV) gleichmäßig über das Bundesgebiet verteilt waren und die die für das Studiendesign passenden Einschlusskriterien (vor allem Fachgruppe) erfüllten. Hausärzte, bei denen kein Patient die Einschlusskriterien der InspeCKD-Studie erfüllte, wurden nicht in die Studie aufgenommen. Alle teilnehmenden Hausärzte nutzen das Arztinformationssystem (AIS) der CompuGroup Medical (CGM) und hatten sich unabhängig von dieser Analyse durch AIS-integrierte Hinweise oder begleitende Informationen bereit erklärt, am Forschungsnetzwerk docmetric der CGM teilzunehmen, das auf die Erfassung und Auswertung realer Versorgungsdaten spezialisiert ist. Dabei werden nur lokal anonymisierte Daten übermittelt.

### Studienpopulation

Gemäß den Screeningempfehlungen von Kidney Disease: Improving Global Outcomes (KDIGO 2020, 2024 [10, 21]) wurden erwachsene CKD-Risikopatienten (ab 18 Jahren), die basierend auf der ICD-10-Codierung an Bluthochdruck und/oder DM und/oder CVD (einschließlich koronarer Herzkrankheit/Atherosklerose, peripherer arterieller Verschlusskrankheit, Myokardinfarkt, Herzrhythmusstörungen, Herzinsuffizienz und Schlaganfall) erkrankt waren, in die Studie eingeschlossen. Für die Analysen wurden lediglich Risikopatienten mit einer individuellen Beobachtungsdauer von mindestens einem Jahr berücksichtigt, was einer endgültigen Studienpopulation von 448.837 Risikopatienten entsprach. Die eGFR wurde mittels Chronic-Kidney-Disease-Epidemiology-Collaboration(CKD-EPI)-2009-Formel berechnet. Nach den Empfehlungen der KDIGO (2012, 2024) wurde eine CKD bei einer Einschränkung der eGFR auf < 60 ml/min pro 1,73 m^2^ oder bei Nachweis einer Albuminurie mit UACR ≥ 30 mg/g, die in ≥ 2 Messungen im Abstand von ≥ 3 Monaten vorlagen, angenommen [10, 22].

### Statistische Auswertung

Alle Analysen wurden explorativ und deskriptiv durchgeführt. Deskriptive Tabellen wurden mit Stichprobenstatistiken erstellt, um demografische und andere Merkmale zusammenzufassen. Kontinuierliche Variablen wurden durch deskriptive Statistiken zusammengefasst, einschließlich Anzahl der Patienten (N), Mittelwert, Standardabweichung, Quartil 1 (Q1), Median, Minimum und Maximum. Kategoriale Variablen wurden durch Häufigkeiten, Prozentsätze und Konfidenzintervalle (KI) dargestellt. Neben der Gesamtpopulation wurden auch präspezifizierte (Sub‑)Populationen untersucht. Alle Analysen wurden mit SAS 9.4 durchgeführt.

## Ergebnisse

### CKD-Risikopatienten

Es wurden Daten von 448.837 Patienten ausgewertet. Das Durchschnittsalter der Patienten zu Studienbeginn betrug 65,1 Jahre, und das Geschlechterverhältnis war ausgeglichen (52,1 % Frauen). Alle eingeschlossenen Patienten hatten aufgrund der zugrunde liegenden Komorbiditäten – 75,8 % Bluthochdruck, 35,1 % kardiovaskuläre Vorerkrankungen (7,9 % Herzinsuffizienz), 32,4 % DM (27,2 % Typ-2-DM) – ein erhöhtes Risiko, eine CKD zu entwickeln, waren also Risikopatienten. Es wiesen 7,5 % der Patienten alle drei Komorbiditäten auf und waren daher als Hochrisikopatienten für eine CKD einzustufen. Weitere Patientencharakteristika sind in Tab. 1 aufgeführt.

**Tab. 1:** Patientencharakteristika

*Gesamtkohorte*	*N* *=* *448.837*
*Demografie*	
Alter in Jahren, Mittelwert (SD)	65,1 (14,5)
< 60 Jahren, *N* (%)	158.070 (35,2)
≥ 60 Jahre, *N* (%)	290.767 (64,8)
Geschlecht, *N* (%)	
Weiblich	233.707 (52,1)
Body-Mass-Index, kg/m^2^, Mittelwert (SD)	29,3 (6,1)
*Anamnese*	
Komorbiditäten, *N* (%)	
Bluthochdruck	340.076 (75,8)
Diabetes mellitus	145.348 (32,4)
Diabetes mellitus Typ 2	121.803 (27,2)
Kardiovaskuläre Erkrankungen*	157.626 (35,1)
Herzinsuffizienz	35.414 (7,9)
Bluthochdruck + Diabetes mellitus + kardiovaskuläre Erkrankungen	33.698 (7,5)
Disease-Management-Programm, *N* (%)	
Diabetes mellitus Typ 2	51.289 (11,4)
Koronare Herzerkrankung	13.644 (3,0)
*Klinische Parameter*	
Blutdruck, mm Hg, Mittelwert (SD)	
Systole	142,2 (21,7)
Diastole	83,6 (12,4)
Lipidstatus, Mittelwert (SD)**	
Cholesterin (Serum), mg/dl	194,6 (47,4)
LDL-Cholesterin, mg/dl	117,1 (41,1)
HDL-Cholesterin, mg/dl	54,4 (16,3)
HbA1c, %, Mittelwert (SD)**	6,8 (1,3)
*Medikation*	
Arzneistoffe mit Wirkung auf das kardiovaskuläre System, *N* (%)	
ACE-Inhibitoren (ACEi)	135.911 (30,3)
Angiotensinrezeptorblocker (ARB)	137.645 (30,7)
Betablocker	166.709 (37,1)
Kalziumkanalblocker	119.014 (26,5)
Diuretika	97.270 (21,7)
Blutzuckersenkende Arzneimittel, *N* (%)	
Dipeptidylpeptidase-4(DPP4)-Hemmer	12.261 (2,7)
Glucagon-like-peptide-1-Rezeptor-Agonisten (GLP-1-RA)	8400 (1,9)
Natrium-Glukose-Kotransporter-2-Hemmer (SGLT-2i)	20.109 (4,5)
Insulin	28.045 (6,3)
Metformin	59.410 (13,2)
Statine, *N* (%)	127.934 (28,5)
Thrombozytenaggregationshemmer, *N* (%)	56.094 (12,5)

*Einschließlich koronarer Herzkrankheit, Atherosklerose, peripherer arterieller Verschlusskrankheit, Myokardinfarkt, Herzinsuffizienz, Herzrhythmusstörungen, Schlaganfall

**Innerhalb der ersten 26 Wochen im Beobachtungszeitraum bei Patienten mit mindestens einer Messung

*ACE* „angiotensin-converting enzyme“, *HbA1c* Hämoglobin A1c, *HDL* High-density-Lipoprotein, *LDL* Low-density-Lipoprotein, *SD* Standardabweichung

### Seltene Diagnose trotz hoher Prävalenz

Die Diagnose CKD kann laborchemisch gestellt werden, wenn im Abstand von ≥ 3 Monaten bei ≥ 2 Serumkreatininmessungen eine eGFR < 60 ml/min pro 1,73 m^2^ vorliegt oder wenn ≥ 2 UACR-Messungen einen Wert ≥ 30 mg/g ergeben [10, 22]. Wie die erste Teilanalyse der InspeCKD-Studie zeigte, wurde bei weniger als der Hälfte (45,5 %) der Risikopatienten mindestens eine Serumkreatininmessung zur Bestimmung der eGFR durchgeführt. Mindestens eine UACR-Bestimmung lag bei 0,4 % der Risikopatienten vor [16]. Die nun vorliegende Analyse zeigt ergänzend, dass bei weniger als einem Drittel der Risikopatienten (28,6 %; *n* = 128.429) die für die CKD-Diagnose erforderliche Labordiagnostik durchgeführt wurde (≥ 2 Serumkreatinin- oder ≥ 2 UACR-Messungen im Abstand von ≥ 3 Monaten). Die CKD-Prävalenz in dieser Studienpopulation betrug 18,8 % (*n* = 24.179). Von diesen Patienten mit erfüllter Krankheitsdefinition erhielten 16,5 % (*n* = 3986) eine CKD-Diagnose nach ICD-10. Die Mehrheit der Patienten (83,5 %; *n* = 20.193) mit erfüllter Krankheitsdefinition blieb ohne CKD-Diagnose nach ICD-10 (Tab. 2).

**Tab. 2:** Prävalenz und Diagnoserate

	Patienten mit für eine CKD-Diagnose ausreichender Labordiagnostik*	Prävalenz (Anteil mit CKD nach KDIGO-Definition** von Patienten mit ausreichender Labordiagnostik)	Diagnoserate (Anteil mit ICD-10-Code für CKD von Patienten mit CKD nach KDIGO-Definition)
	*N* (%)	*N* (%)	*n*/*N* (%)
Gesamtkohorte	128.429 (28,6)	24.179 (18,8)	3986/24.179 (16,5)
*Stratifiziert nach Komorbiditäten*			
Bluthochdruck	87.603 (25,8)	15.907 (18,2)	2132/15.907 (13,4)
Diabetes mellitus	61.820 (42,5)	12.165 (19,7)	2458/12.165 (20,2)
Diabetes mellitus Typ 2	53.014 (43,5)	10.501 (19,8)	2187/10.501 (20,8)
Kardiovaskuläre Erkrankungen	53.592 (34,0)	12.927 (24,1)	2167/12.927 (20,8)
Herzinsuffizienz	12.257 (34,6)	3761 (30,7)	683/3761 (18,2)
Bluthochdruck + Diabetes mellitus + kardiovaskuläre Erkrankungen	15.025 (44,6)	3756 (25,0)	653/3756 (17,4)
*Stratifiziert nach ausgewählten (Sub‑)Populationen*			
Geschlecht			
Männlich	61.362 (28,5)	9373 (15,3)	1790/9373 (19,1)
Weiblich	67.048 (28,7)	14.806 (22,1)	2196/14.806 (14,8)
Alter			
< 60 Jahren	35.047 (22,2)^§^	1079 (3,1)^§^	171/910 (18,8)^†^
≥ 60 Jahre	93.382 (32,1)^§^	23.100 (24,7)^§^	3815/23.269 (16,4)^†^

*≥ 2 eGFR- oder ≥ 2 UACR-Messungen im Abstand von ≥ 3 Monaten

**Anteil der Patienten mit CKD nach KDIGO-Definition (eGFR < 60 ml/min pro 1,73 m^2^ bzw. UACR > 30 mg/g in ≥ 2 Messungen im Abstand von ≥ 3 Monaten) bei Patienten mit ausreichender Labordiagnostik

^§^Zu Studienbeginn

^†^Zum Zeitpunkt der Diagnose

*CKD* „chronic kidney disease“ (chronische Nierenerkrankung), *eGFR* geschätzte glomeruläre Filtrationsrate, *ICD-10* Internationale statistische Klassifikation der Krankheiten und verwandter Gesundheitsprobleme, *KDIGO* Kidney Disease: Improving Global Outcomes, *UACR* „urine albumine-creatinine ratio“ (Albumin-Kreatinin-Verhältnis)

Die Analysen der einzelnen (Sub‑)Populationen zeigten, dass bei jedem zweiten Risikopatienten mit DM (42,5 %, *n* = 61.820), bei jedem dritten Risikopatienten mit CVD (34,0 %, *n* = 53.592) und bei jedem vierten Risikopatienten mit Bluthochdruck (25,8 %, *n* = 87.303) Labordiagnostik vorlag (≥ 2 eGFR- oder ≥ 2 UACR-Messungen im Abstand von ≥ 3 Monaten), um eine CKD-Diagnose stellen zu können. Die CKD-Prävalenz betrug 18,2 % (*n* = 15.907) bei Bluthochdruck, 19,7 % (*n* = 12.165) bei DM und 24,1 % (*n* = 12.927) bei CVD. Bei Risikopatienten mit Herzinsuffizienz war die CKD-Prävalenz mit 30,7 % (*n* = 3761) höher. Auch jeder vierte Hochrisikopatient (25,0 %; *n* = 3756) erfüllte die CKD-Definition nach KDIGO. Der Anteil der Patienten mit erfüllter Krankheitsdefinition, bei denen auch eine Diagnose nach ICD-10 dokumentiert wurde, war mit jeweils 20,8 % bei CVD und Typ-2-DM am höchsten. Hochrisikopatienten (17,4 %) und insbesondere Patienten mit Bluthochdruck (13,4 %) wurden seltener nach ICD-10 diagnostiziert (Tab. 2).

### Ältere Patienten verbleiben häufiger ohne CKD-Diagnose nach ICD-10

Die Analyse in Bezug auf das Alter ergab, dass ältere Patienten (≥ 60 Jahre) mit einer Prävalenz zu Studienbeginn von 24,7 % (*n* = 23.100) ein 8‑mal höheres Risiko für das Vorliegen einer CKD haben als jüngere Patienten (3,1 %; *n* = 1079). Auffallend war, dass bei älteren Patienten (Alter zum Zeitpunkt der Diagnose; 16,4 %) im Vergleich zu jüngeren Patienten (18,8 %) seltener eine CKD-Diagnose nach ICD-10 gestellt und dokumentiert wurde (Tab. 2).

### CKD bei Frauen seltener nach ICD-10 diagnostiziert und schlechter behandelt

Bei der Analyse wurden auch Unterschiede zwischen den Geschlechtern deutlich. Obwohl die Prävalenz der CKD bei Frauen mit 22,1 % um das 1,5-fache höher als bei Männern war (CKD-Prävalenz 15,3 %), wurde bei Frauen seltener eine CKD nach ICD-10 diagnostiziert (14,8 %; *n* = 2196 vs. 19,1 %; *n* = 1790 bei Männern; Tab. 2). Frauen wurden zudem seltener mit der für die meisten Patienten empfohlenen Therapie (RASi in Kombination mit einem SGLT-2i) behandelt (7,3 % gegenüber 12,1 % der Männer; Tab. 5).

### CKD-Patienten mit und ohne CKD-Diagnose nach ICD-10 unterscheiden sich kaum

In den nachfolgenden Analysen wurden ausschließlich Risikopatienten mit CKD berücksichtigt, die in folgende Gruppen unterteilt wurden:

- Risikopatienten mit einer nach ICD-10 dokumentierten CKD-Diagnose (nachfolgend „CKD-Patienten mit ICD-10-Diagnose“ genannt)
- Risikopatienten, die die CKD-Definition erfüllen (≥ 2 eGFR- oder ≥ 2 UACR-Messungen im Abstand von ≥ 3 Monaten), bei denen jedoch keine CKD-Diagnose nach ICD-10 dokumentiert wurde (nachfolgend „CKD-Patienten ohne ICD-10-Diagnose“ genannt)

CKD-Patienten ohne ICD-10-Diagnose waren im Durchschnitt 4 Jahre älter und weniger häufig an Bluthochdruck und CVD (einschließlich Herzinsuffizienz) erkrankt als CKD-Patienten mit ICD-10-Diagnose. Ein DM war dagegen in beiden Gruppen etwa gleich häufig. Hinsichtlich klinischer Parameter konnten keine wesentlichen Unterschiede zwischen den beiden Gruppen festgestellt werden (Tab. 3).

**Tab. 3:** Übersicht über Patienten mit und ohne diagnostizierte CKD (ICD-10)

	CKD-Patienten mit ICD-10-Diagnose	CKD-Patienten ohne ICD-10-Diagnose
	*N* = 17.952	*N* = 14.199
***Demografie***		
**Alter, Jahre**		
Mittelwert (SD)	74,7 (11,5)	78,3 (8,8)
95 %-KI	74,6–74,9	78,2–78,5
**Männlich**		
*N* (%)	8796 (49,0)	5315 (37,4)
95 %-KI	48,3–49,7	36,6–38,2
**Weiblich**		
*N* (%)	9154 (51,0)	8884 (62,6)
95 %-KI	50,3–51,7	61,8–63,4
Body-Mass-Index, kg/m^2^		
Mittelwert (SD)	29,4 (5,8)	29,3 (6,3)
95 %-KI	29,0–30,0	29,0–30,0
***Anamnese***		
Komorbiditäten*		
*Bluthochdruck*		
*N* (%)	15.172 (84,5)	10.226 (72,0)
95 %-KI	84,0–85,0	71,3–72,8
*Diabetes mellitus*		
*N* (%)	10.173 (56,7)	7721 (54,4)
95 %-KI	55,9–57,4	53,6–55,2
*Diabetes mellitus Typ 2*		
*N* (%)	9277 (51,7)	6723 (47,4)
95 %-KI	50,9–52,4	46,5–48,2
*Kardiovaskuläre Erkrankungen*		
*N* (%)	11.296 (62,9)	8267 (58,2)
95 %-KI	62,2–63,6	57,4–59,0
*Herzinsuffizienz*		
*N* (%)	4469 (24,9)	2585 (18,2)
95 %-KI	24,3–25,5	17,6–18,9
Bluthochdruck + Diabetes mellitus + kardiovaskuläre Erkrankungen		
*N* (%)	5339 (29,7)	2974 (21,0)
95 %-KI	29,1–30,4	20,3–21,6
***Klinische Parameter***		
**Blutdruck, mm** **Hg**		
*Systole*		
*N* (%)	139,3 (21,7)	137,6 (20,6)
95 %-KI	138,0–140,0	137,0–138,0
*Diastole*		
*N* (%)	79,8 (11,9)	78,1 (11,2)
95 %-KI	79,0–80,0	78,0–79,0

*Zwischen Beginn des Beobachtungszeitraums und Indexdatum

*CKD* „chronic kidney disease“ (chronische Nierenerkrankung), *ICD-10* Internationale statistische Klassifikation der Krankheiten und verwandter Gesundheitsprobleme, *KI* Konfidenzintervall, *SD* Standardabweichung

### CKD-Patienten kaum nach aktueller Leitlinienempfehlung behandelt

Die Analyse der Behandlung von CKD-Patienten 6 Monate nach Diagnosestellung bzw. Erfüllung der Krankheitskriterien ergab, dass ein geringer Anteil der CKD-Patienten eine an die CKD angepasste medikamentöse Therapie erhielt. Die für die meisten Patienten empfohlene Therapie mit einem RASi in Kombination mit einem SGLT-2i erhielten 9,7 % (*n* = 1740) der CKD-Patienten mit ICD-10-Diagnose und 10,3 % (*n* = 1459) der CKD-Patienten ohne ICD-10-Diagnose (Tab. 4). Auch innerhalb der analysierten (Sub‑)Populationen wurde die für die meisten Patienten empfohlene Therapie mit einem RASi in Kombination mit einem SGLT-2i nicht häufiger eingesetzt (Tab. 4 und 5). Lediglich bei Hochrisikopatienten (16,5 % der CKD-Patienten mit ICD-10-Diagnose bzw. 16,6 % der CKD-Patienten ohne ICD-10-Diagnose) und bei Patienten mit Typ-2-DM (15,3 % der CKD-Patienten mit ICD-10-Diagnose bzw. 15,8 % der CKD-Patienten ohne ICD-10-Diagnose) wurde diese Therapie etwas häufiger eingesetzt (Tab. 4). Zur Behandlung der CKD wurden in erster Linie RASi und Statine eingesetzt. Eine SGLT-2i-Therapie erhielt jeder achte CKD-Risikopatient (12,5 % der CKD-Patienten mit ICD-10-Diagnose; 12,7 % der CKD-Patienten ohne ICD-10-Diagnose; Tab. 4), wobei ein SGLT-2i häufiger bei Typ-2-DM (21,0 % der CKD-Patienten mit ICD-10-Diagnose; 20,0 % der CKD-Patienten ohne ICD-10-Diagnose) eingesetzt wurde. Im Vergleich dazu wurden CKD-Patienten ohne Typ-2-DM seltener mit SGLT-2i (5,8 % der CKD-Patienten mit ICD-10-Diagnose; 6,8 % der CKD-Patienten ohne ICD-10-Diagnose) behandelt. Auch Hochrisikopatienten wurden häufiger mit SGLT-2i (21,7 % der CKD-Patienten mit ICD-10-Diagnose bzw. 19,4 % der CKD-Patienten ohne ICD-10-Diagnose) behandelt (Tab. 4).

**Tab. 4:** CKD-Therapie innerhalb von 6 Monaten*

	CKD-Patienten mit ICD-10-Diagnose		CKD-Patienten ohne ICD-10-Diagnose	
	*N* (%)	95 %-KI	*N* (%)	95 %-KI
Gesamtkohorte	*N* = 17.952		*N* = 14.199	
RASi**	13.259 (73,9)	73,2–74,5	10.979 (77,3)	76,6–78,0
Statine	9154 (51,0)	50,3–51,7	7565 (53,3)	52,5–54,1
SGLT-2i	2252 (12,5)	12,1–13,0	1797 (12,7)	12,1–13,2
RASi + SGLT-2i	1740 (9,7)	9,3–10,1	1459 (10,3)	9,8–10,8
RASi + SGLT-2i + Statine	1222 (6,8)	6,4–7,2	1015 (7,2)	6,7–7,6
**Stratifiziert nach Komorbiditäten**				
*Bluthochdruck*	*N* *=* *11.653*		*N* *=* *9445*	
RASi	9079 (77,9)	77,2–78,7	7712 (81,7)	80,9–82,4
Statine	5621 (48,2)	47,3–49,2	4858 (51,4)	50,4–52,5
SGLT-2i	1264 (10,9)	10,3–11,4	1001 (10,6)	10,0–11,2
RASi + SGLT-2i	991 (8,5)	8,0–9,0	856 (9,1)	8,5–9,7
RASi + SGLT-2i + Statine	669 (5,7)	5,3–6,2	571 (6,1)	5,6–6,6
*Herzinsuffizienz*	*N* *=* *2882*		*N* *=* *2203*	
RASi	2196 (76,2)	74,6–77,7	1778 (80,7)	79,0–82,3
Statine	1560 (54,1)	52,3–56,0	1173 (53,3)	51,1–55,4
SGLT-2i	471 (16,3)	15,0–17,4	374 (17,0)	15,4–18,6
RASi + SGLT-2i	407 (14,1)	12,9–15,5	328 (14,9)	13,4–16,4
RASi + SGLT-2i + Statine	286 (9,9)	8,9–11,1	225 (10,2)	9,0–11,6
*Kardiovaskuläre Erkrankungen*	*N* *=* *9057*		*N* *=* *7652*	
RASi	6795 (75,0)	74,1–75,9	5950 (77,8)	76,8–78,7
Statine	5489 (60,6)	59,6–61,6	4646 (60,7)	59,6–61,8
SGLT-2i	1236 (13,7)	13,0–14,4	1001 (13,1)	12,3–13,9
RASi + SGLT-2i	999 (11,0)	10,4–11,7	863 (11,3)	10,6–12,0
RASi + SGLT-2i + Statine	740 (8,2)	7,6–8,8	643 (8,4)	7,8–9,1
*Diabetes mellitus Typ 2*	*N* *=* *7975*		*N* *=* *6328*	
RASi	5702 (71,5)	70,5–72,5	4833 (76,4)	75,3–77,4
Statine	4346 (54,5)	53,4–55,6	3457 (54,6)	53,4–55,9
SGLT-2i	1673 (21,0)	20,1–21,9	1263 (20,0)	19,0–21,0
RASi + SGLT-2i	1218 (15,3)	14,5–16,1	998 (15,8)	14,9–16,7
RASi + SGLT-2i + Statine	858 (10,8)	10,1–11,5	685 (10,8)	10,1–11,6
*Kein Diabetes mellitus Typ 2*	*N* *=* *9977*		*N* *=* *7871*	
RASi	7557 (75,7)	74,9–76,6	6146 (78,1)	77,2–79,0
Statine	4808 (48,2)	47,2–49,2	4108 (52,2)	51,1–53,3
SGLT-2i	579 (5,8)	5,4–6,3	534 (6,8)	6,2–7,4
RASi + SGLT-2i	522 (5,2)	4,8–5,7	461 (5,9)	5,4–6,4
RASi + SGLT-2i + Statine	364 (3,7)	3,3–4,0	330 (4,2)	3,8–4,7
*Bluthochdruck* *+* *Diabetes mellitus* *+* *kardiovaskuläre Erkrankungen*	*N* *=* *2650*		*N* *=* *2336*	
RASi	2013 (76,0)	74,3–77,6	1905 (81,6)	79,9–83,1
Statine	1642 (62,0)	60,1–63,8	1478 (63,3)	61,3–65,2
SGLT-2i	574 (21,7)	20,1–23,3	452 (19,4)	17,8–21,0
RASi + SGLT-2i	438 (16,5)	15,1–18,0	387 (16,6)	15,1–18,1
RASi + SGLT-2i + Statine	326 (12,3)	11,1–13,6	289 (12,4)	11,1–13,8

*Zwischen Beginn des Beobachtungszeitraums und 26 Wochen nach Indexdatum

**ACE-Inhibitoren oder Angiotensinrezeptorblocker

*ACE* „angiotensin-converting enzyme“, *CKD* „chronic kidney disease“ (chronische Nierenerkrankung), *ICD-10* Internationale statistische Klassifikation der Krankheiten und verwandter Gesundheitsprobleme, *KI* Konfidenzintervall, *RASi* Hemmer des Renin-Angiotensin-Systems, *SGLT-2i* Hemmer des Natrium-Glukose-Kotransporters 2

**Tab. 5:** CKD-Therapie innerhalb von 6 Monaten* in ausgewählten (Sub‑)Populationen

	CKD-Patienten mit ICD-10-Diagnose		CKD-Patienten ohne ICD-10-Diagnose	
	*N* (%)	95 %-KI	*N* (%)	95 %-KI
**Stratifiziert nach Alter**				
*<* *60 Jahren*	*N* *=* *2074*		*N* *=* *503*	
RASi	1354 (65,3)	63,2–67,3	357 (71,0)	66,8–74,9
Statine	734 (35,4)	33,3–37,5	206 (41,0)	36,6–45,4
SGLT-2i	284 (13,7)	12,2–15,3	87 (17,3)	14,1–20,9
RASi + SGLT-2i	193 (9,3)	8,1–10,6	71 (14,1)	11,2–17,5
RASi + SGLT-2i + Statine	117 (5,6)	4,7–6,7	43 (8,6)	6,3–11,3
*≥* *60 Jahre*	*N* *=* *15.878*		*N* *=* *13.696*	
RASi	11.905 (75,0)	74,3–75,7	10.622 (77,6)	76,9–78,3
Statine	8420 (53,0)	52,25–53,81	7359 (53,7)	52,9–54,6
SGLT-2i	1968 (12,4)	11,9–12,9	1710 (12,5)	11,9–13,1
RASi + SGLT-2i	1547 (9,7)	9,3–10,2	1388 (10,1)	9,6–10,7
RASi + SGLT-2i + Statine	1105 (7,0)	6,6–7,4	972 (7,1)	6,7–7,5
**Stratifiziert nach Geschlecht**				
*Männlich*	*N* *=* *8796*		*N* *=* *5315*	
RASi	6573 (74,7)	73,8–75,6	4132 (77,7)	76,6–78,9
Statine	4891 (55,6)	54,6–56,7	3211 (60,4)	59,1–61,7
SGLT-2i	1364 (15,5)	14,8–16,3	958 (18,0)	17,0–19,1
RASi + SGLT-2i	1067 (12,1)	11,5–12,8	775 (14,6)	13,6–15,6
RASi + SGLT-2i + Statine	770 (8,8)	8,2–9,4	573 (10,8)	10,0–11,7
*Weiblich*	*N* *=* *9154*		*N* *=* *8884*	
RASi	6685 (73,0)	72,1–73,9	6847 (77,1)	76,2–77,9
Statine	4262 (46,6)	45,5–47,6	4354 (49,0)	48,0–50,1
SGLT-2i	887 (9,7)	9,1–10,3	839 (9,4)	8,8–10,1
RASi + SGLT-2i	672 (7,3)	6,8–7,9	684 (7,7)	7,2–8,3
RASi + SGLT-2i + Statine	452 (4,9)	4,5–5,4	442 (5,0)	4,5–5,5
**Stratifiziert nach eGFR-Kategorie der vorliegenden CKD**				
*G1 – normal oder erhöht (≥* *90* *ml/min pro 1,73* *m*^*2*^*)*	*N* *=* *204*		*N* *=* *15*	
RASi	131 (64,2)	57,2–70,8	9 (60,0)	32,3–83,7
Statine	95 (46,6)	39,6–53,7	3 (20,0)	4,3–48,1
SGLT-2i	57 (27,9)	21,9–34,6	10 (66,7)	38,4–88,2
RASi + SGLT-2i	38 (18,6)	13,5–24,7	7 (46,7)	21,3–73,4
RASi + SGLT-2i + Statine	26 (12,8)	8,5–18,1	2 (13,3)	1,7–40,5
*G2 – leicht vermindert (60–<* *90* *ml/min pro 1,73* *m*^*2*^*)*	*N* *=* *1473*		*N* *=* *25*	
RASi	1041 (70,7)	68,3–73,0	18 (72,0)	50,6–87,9
Statine	770 (52,3)	49,7–54,9	14 (56,0)	34,9–75,6
SGLT-2i	207 (14,1)	12,3–15,9	5 (20,0)	6,8–40,7
RASi + SGLT-2i	144 (9,8)	8,3–11,4	3 (12,0)	2,6–31,2
RASi + SGLT-2i + Statine	102 (6,9)	5,7–8,3	3 (12,0)	2,6–31,2
*G3a – leicht bis mäßig vermindert (45–<* *60* *ml/min pro 1,73* *m*^*2*^*)*	*N* *=* *2151*		*N* *=* *9424*	
RASi	1596 (74,2)	72,3–76,0	7192 (76,3)	75,4–77,2
Statine	1174 (54,6)	52,5–56,7	5034 (53,4)	52,4–54,4
SGLT-2i	334 (15,5)	14,0–17,1	1178 (12,5)	11,8–13,2
RASi + SGLT-2i	235 (10,9)	9,6–12,3	943 (10,0)	9,4–10,6
RASi + SGLT-2i + Statine	167 (7,8)	6,7–9,0	670 (7,1)	6,6–7,7
*G3b – mäßig bis stark vermindert (30–<* *45* *ml/min pro 1,73* *m*^*2*^*)*	*N* *=* *1867*		*N* *=* *3710*	
RASi	1463 (78,4)	76,4–80,2	2977 (80,2)	78,9–81,5
Statine	1022 (54,7)	52,5–57,0	1969 (53,1)	51,5–54,7
SGLT-2i	311 (16,7)	15,0–18,4	483 (13,0)	12,0–14,1
RASi + SGLT-2i	237 (12,7)	11,2–14,3	401(10,8)	9,8–11,9
RASi + SGLT-2i + Statine	169 (9,1)	7,8–10,5	266 (7,2)	6,4–8,1
*G4 – stark vermindert (15–<* *30* *ml/min pro 1,73* *m*^*2*^*)*	*N* *=* *755*		*N* *=* *737*	
RASi	612 (81,1)	78,1–83,8	573 (77,8)	74,6–80,7
Statine	408 (54,0)	50,4–57,6	396 (53,7)	50,1–57,4
SGLT-2i	117 (15,5)	13,0–18,3	86 (11,7)	9,4–14,2
RASi + SGLT-2i	94 (12,5)	10,2–15,0	73 (9,9)	7,8–12,3
RASi + SGLT-2i + Statine	63 (8,3)	6,5–10,6	51 (6,9)	5,2–9,0
*G5 – Nierenversagen (<* *15* *ml/min pro 1,73* *m*^*2*^*)*	*N* *=* *114*		*N* *=* *280*	
RASi	76 (66,7)	57,2–75,2	207 (73,9)	68,4–79,0
Statine	53 (46,5)	37,1–56,1	145 (51,8)	45,8–57,8
SGLT-2i	10 (8,8)	4,3–15,5	35 (12,5)	8,9–17,0
RASi + SGLT-2i	9 (7,9)	3,7–14,5	32 (11,4)	8,0–15,8
RASi + SGLT-2i + Statine	6 (5,3)	2,0–11,1	23 (8,2)	5,3–12,1

*Zwischen Beginn des Beobachtungszeitraums und 26 Wochen nach Indexdatum

**ACE-Inhibitoren oder Angiotensinrezeptorblocker

*ACE* „angiotensin-converting enzyme“, *CKD* „chronic kidney disease“ (chronische Nierenerkrankung), *eGFR* geschätzte glomeruläre Filtrationsrate, *ICD-10* Internationale statistische Klassifikation der Krankheiten und verwandter Gesundheitsprobleme, *KI* Konfidenzintervall, *RASi* Hemmer des Renin-Angiotensin-Systems, *SGLT-2i* Hemmer des Natrium-Glukose-Kotransporters 2

## Diskussion

Nach Untersuchung der Labordiagnostik in der ersten Teilanalyse [16] legte die vorliegende zweite Teilanalyse der InspeCKD-Studie den Fokus auf Prävalenz, Diagnostik, Diagnose und Therapie der CKD bei Risikopatienten. Die Daten zeigen in Abhängigkeit von Komorbiditäten, Alter und Geschlecht Defizite bei der Diagnostik, Diagnose und Behandlung von CKD in der hausärztlichen Routineversorgung in Deutschland auf.

### Prävalenz und Diagnosehäufigkeit

Leitlinien sollen Ärzten patientenorientierte Empfehlungen für die Diagnostik, Diagnose und Therapie geben. In Anlehnung an die nationalen VersorgungsLeitlinien sollte die Nierenfunktion bei Risikopatienten regelmäßig überwacht werden [13–15], um die mit CKD verbundenen Gesundheitsrisiken reduzieren zu können. So haben beispielsweise Patienten mit DM ein erhöhtes Risiko, eine diabetische Nephropathie zu entwickeln. Auch ein erhöhter Blutdruck ist mit einem erhöhten Risiko kardiovaskulärer und renaler Komplikationen verbunden [23]. Wie die InspeCKD-Studie aufzeigt, beträgt die Prävalenz der CKD bei Risikopatienten in Deutschland 18,8 %. Umso erstaunlicher ist es, dass 5 von 6 Risikopatienten (83,5 %) nicht nach ICD-10 diagnostiziert werden, obwohl bei ihnen die Definition der CKD erfüllt ist (≥ 2-mal eGFR < 60 ml/min pro 1,73 m^2^ oder UACR > 30 mg/g im Abstand von ≥ 3 Monaten; [10, 22]). Eine weitere deutschlandweit durchgeführte Studie zeigte, dass bei etwa 80 % der Patienten mit CKD die prävalente Niereninsuffizienz nicht in der Patientenakte dokumentiert war [6]. Wie die Daten der InspeCKD-Studie belegen, ist die Dunkelziffer in frühen Stadien besonders hoch: 91,8 % der Risikopatienten im Stadium G3a und 78,2 % der Risikopatienten im Stadium G3b wurden nicht nach ICD-10 diagnostiziert. Mängel bei der Diagnose von CKD im Stadium 3 wurden bereits in der REVEAL-CKD-Studie festgestellt [4]. Ein beträchtlicher Anteil der nicht nach ICD-10 diagnostizierten CKD-Fälle könnte möglicherweise auf einer fehlenden Incentivierung für die Hausärzte beruhen, eine CKD nach ICD-10 zu codieren. Dennoch sollte eine CKD-Diagnose von einer ICD-10-Codierung begleitet sein und in der Patientenakte vermerkt werden.

Der hohe Anteil nicht nach ICD-10 diagnostizierter CKD bei hohem CKD-Risiko ist besorgniserregend

Der hohe Anteil nicht nach ICD-10 diagnostizierter CKD trotz vorliegender Labordiagnostik bei Patienten mit hohem CKD-Risiko ist besorgniserregend, da diese Patienten ein erhöhtes Risiko für eine CKD-Progression und damit verbundene Komplikationen haben. Eine nicht diagnostizierte und therapierte CKD trägt zu einer hohen Kosten- und Versorgungslast in der Primär- und Sekundärversorgung bei. Die in den Nationalen VersorgungsLeitlinien formulierten Empfehlungen zur regelmäßigen Kontrolle der Nierenfunktion bei Risikopatienten [13, 14] scheinen nicht zu einer ICD-10-gestützten Diagnosestellung zu führen. Dabei kann ein erhöhtes Bewusstsein für CKD bei Hausärzten zu einer verbesserten und frühzeitigen Diagnose von CKD führen [24].

Obwohl die Prävalenz der CKD in unserer Analyse mit dem Alter zunahm, war der Anteil der nicht nach ICD-10 diagnostizierten CKD bei älteren Patienten (≥ 60 Jahre) höher, was bedeutet, dass ältere Patienten ein höheres Risiko für eine verzögerte Diagnose haben als jüngere Patienten (< 60 Jahren). Dies ist beunruhigend, da altersbedingte physiologische Veränderungen der Pharmakodynamik in Verbindung mit einer Abnahme der glomerulären Filtrationsrate eine regelmäßige Überwachung der Nierenfunktion und gegebenenfalls eine Anpassung der Verschreibung und Dosierung von Medikamenten erfordern [25]. Es ist daher wichtig, dass Hausärzte einerseits die physiologisch bedingte Abnahme der Nierenfunktion (GFR) nicht notgedrungen als CKD deuten, andererseits aber die erhöhte Vulnerabilität älterer Patienten nicht unterschätzen und diese Patienten bei Bedarf ebenfalls nach den aktuellen Leitlinienempfehlungen behandeln.

Obwohl Frauen häufiger an einer CKD erkranken, erhalten sie seltener eine CKD-Diagnose und seltener eine CKD-Therapie

Frauen haben ein höheres Risiko, an einer CKD zu erkranken und CKD-bedingte gesundheitliche Folgen, wie kardiovaskuläre Ereignisse und vorzeitige Mortalität, zu erleiden [26, 27]. Analog dazu zeigte die InspeCKD-Studie eine erhöhte Prävalenz der CKD bei Frauen (22,1 % vs. 15,3 % bei Männern). Obwohl Frauen häufiger an einer CKD erkranken, haben Männer ein höheres Risiko für Nierenversagen [28], Männer erhalten zudem häufiger eine Nierenersatztherapie als Frauen [29, 30]. Diese Unterschiede könnten womöglich darauf zurückzuführen sein, dass die Erkrankung bei Frauen langsamer voranschreitet [31, 32], Frauen sich häufiger gegen eine Dialyse aussprechen [33] und mutmaßlich Frauen unverhältnismäßig häufig aufgrund anderer Ursachen sterben, bevor eine Nierenersatztherapie in Betracht gezogen wird [34]. Frauen, die sich einer Dialyse unterzogen, wiesen eine höhere Hospitalisierungsrate auf als Männer [35].

Trotz der hohen Prävalenz der CKD wurden Frauen seltener nach ICD-10 diagnostiziert (etwa jede siebte Frau; 14,8 %) als Männer (etwa jeder fünfte Mann; 19,1 %). Internationale Studien zeigten ebenfalls geschlechtsspezifische Unterschiede in der Diagnosehäufigkeit [5, 36–39]. Außerdem erhielten Frauen (7,3 %) seltener als Männer (12,1 %) eine CKD-Therapie mit einem RASi in Kombination mit einem SGLT-2i. Dies deutet auf eine Unterversorgung von Frauen hinsichtlich der CKD in der hausärztlichen Versorgung auch in Deutschland hin und stützt die Daten internationaler Studien, die erhebliche geschlechtsspezifische Unterschiede in Diagnosestellung, Monitoring und Management der CKD bei Frauen zeigen [37, 38, 40].

Die Daten der InspeCKD-Studie zeigen zudem, dass die CKD-Diagnose nach ICD-10 in frühen Stadien der Erkrankung, das heißt bei einer zum Zeitpunkt der Diagnose leicht verminderten eGFR von 60 bis 90 ml/min pro 1,73 m^2^ (CKD-Stadium G2), durchaus auch auf Grundlage der UACR gestellt wird (die geringe Anzahl an Patienten lässt nur eine eingeschränkte Auswertung zu) und dass bei diesen Patienten die Diagnosestellung stringenter erfolgt als auf Grundlage der eGFR. Dies unterstreicht die Relevanz der UACR-Bestimmung als einen entscheidenden Früherkennungsmarker für eine CKD.

### Medikamentöse Therapie der CKD

Frühzeitige Interventionen bei CKD verbessern nachweislich das Behandlungsergebnis, indem sie das Fortschreiten der CKD verlangsamen und das kardiovaskuläre Risiko senken [21]. Über viele Jahrzehnte hinweg konnten neben der RAS-Blockade keine neuen Therapien in den klinischen Alltag eingeführt werden, um die Progression der CKD aufzuhalten.

Wie klinische Studien zeigen, führen SGLT-2i zu einer signifikanten Verringerung der kardiovaskulären und nierenbedingten Folgeschäden und der Gesamtmortalität [20]. Patienten können somit möglichst lange vor einer terminalen Niereninsuffizienz bewahrt werden. Für die Behandlung von Erwachsenen mit CKD gibt die KDIGO in ihrer aktuellen Leitlinie von 2024 eine allgemeinere 1A-Empfehlung, die nicht mehr allein auf CKD-Patienten mit Typ-2-DM beschränkt ist. Eine Therapie mit einem SGLT-2i kann daher bei den meisten Patienten mit CKD und einer eGFR ≥ 20 ml/min pro 1,73 m^2^ und einer UACR ≥ 200 mg/g unabhängig von einem Typ-2-DM angeraten sein, wobei eine Kombination mit einem RASi erwogen werden kann [10]. Die KDIGO hebt damit die Bedeutung der SGLT-2i bei CKD-Patienten hinsichtlich der Verlangsamung der CKD-Progression sowie hinsichtlich der Verringerung des Risikos eines Nierenversagens, der kardiovaskulären Mortalität und einer Herzinsuffizienz hervor.

Die Leitlinie des National Institute for Health and Care Excellence (NICE) empfiehlt SGLT-2i (Dapagliflozin) als Add-on-Therapie zur Behandlung der CKD für Patienten mit einer eGFR von 25 bis 75 ml/min pro 1,73 m^2^, die bereits einen RASi (Angiotensin-converting-enzyme[ACE]-Hemmer oder Angiotensinrezeptorblocker) erhalten und einen Typ-2-DM oder eine UACR ≥ 200 mg/g aufweisen [41].

Die aktualisierte S3-Leitlinie der Deutschen Gesellschaft für Allgemeinmedizin und Familienmedizin (DEGAM) von 2024 zur „Versorgung von Patient*innen mit chronischer, nicht-nierenersatztherapiepflichtiger Nierenkrankheit in der Hausarztpraxis“ empfiehlt hingegen die Therapie mit einem SGLT-2i bei Patienten mit einer Albuminurie ≥ 300 mg/g und/oder einer eGFR < 45 ml/min pro 1,73 m^2^ (Empfehlung 8.11; [15]). Damit individuell eine differenzierte Entscheidung zur Einnahme eines SGLT-2i bei Patienten mit CKD getroffen werden kann, bietet die DEGAM eine Orientierungshilfe an, in der verschiedene Aspekte aufgeführt werden, die bei der Indikationsstellung berücksichtigt werden sollten [15].

Die empfohlene Erstlinientherapie mit RASi und SGLT-2i findet zu selten Anwendung

Die Daten der InspeCKD-Studie zeigen, dass nur jeder achte CKD-Patient (12,5 %) in der hausärztlichen Versorgung binnen 6 Monaten nach Diagnosestellung mit einem SGLT-2i behandelt wurde. Die empfohlene Erstlinientherapie mit einem RASi in Kombination mit einem SGLT-2i findet ebenfalls zu selten Anwendung (9,7 % der Risikopatienten). Eine unzureichende Diagnosestellung kann mitunter dazu führen, dass Möglichkeiten zur Verschreibung neuer Therapieansätze (beispielsweise SGLT-2i-Therapie), die nachweislich das Fortschreiten der CKD verlangsamen und das kardiovaskuläre Risiko senken, seltener Anwendung finden [12, 21]. Dennoch unterschied sich die CKD-Therapie unwesentlich zwischen Patienten mit nach ICD-10 diagnostizierter CKD und Patienten ohne CKD-Diagnose nach ICD-10. Folglich bleibt eine CKD-Diagnose nach ICD-10 trotz empfohlener Behandlungsoption aktuell noch ohne relevanten Einfluss auf die CKD-Therapie bzw. das CKD-Management und erfolgt mutmaßlich nicht vordergründig aufgrund der CKD. Der nach wie vor verbreitetere Einsatz von RASi (73,9 %) für die CKD-Therapie kann zum einen darauf zurückzuführen sein, dass sie die Basistherapie bei Patienten mit Bluthochdruck darstellen (75,8 % der Risikopatienten hatten Bluthochdruck) und daher nicht spezifisch aufgrund der CKD als Behandlungsoption gewählt werden. Zum anderen kann es daran liegen, dass neuere CKD-Therapieoptionen wie die SGLT-2i noch nicht im Bewusstsein deutscher Hausärzte etabliert sind und daher noch nicht in Betracht gezogen werden. Da frühzeitige therapeutische Interventionen bei CKD das Fortschreiten der Erkrankung verlangsamen, das kardiovaskuläre Risiko verringern und somit die mit der Erkrankung verbundenen Gesundheitskosten reduzieren [7, 21], ist es insbesondere für Hausärzte von entscheidender Bedeutung, die Erkrankung so früh wie möglich zu diagnostizieren und zu behandeln. Auf diese Weise können Folgeerkrankungen der CKD und damit verbundene Einschränkungen der Lebensqualität reduziert werden.

### Limitationen

Die erhobenen Sekundärdaten stammen aus der Routineversorgung und können Verzerrungen hinsichtlich der Repräsentativität der Hausarztpraxen und/oder der Patienten unterliegen. Daten aus dem Krankenhaussektor und/oder aus fachärztlichen Praxen sind hingegen nicht enthalten. Eventuell dort gestellte Diagnosen, die nicht in das hausärztliche Arztinformationssystem übertragen wurden, werden somit nicht abgebildet. Die an der Studie beteiligten Hausärzte und hausärztlich tätigen Internisten waren regional ausgewogen verteilt. Trotz sorgfältiger Prüfung können Dokumentationsunterschiede in den Hausarztpraxen die Datenqualität und -vollständigkeit beeinflussen. Dennoch ist dieser außergewöhnlich große Datensatz mit Daten von 1244 deutschen Hausärzten und fast einer halben Million analysierter Patienten aus ganz Deutschland, die ein breites Spektrum relevanter Komorbiditäten repräsentieren, eine wertvolle Ergänzung der bisher verfügbaren Evidenz. Als unzureichend muss der Anteil der Risikopatienten mit einer Metformintherapie bei einem Anteil der Patienten mit Diabetes von 32,4 % erachtet werden.

### Resümee

Die InspeCKD-Studie verdeutlicht, dass ein großer Teil der CKD-Risikopatienten in der hausärztlichen Routineversorgung in Deutschland nicht auf das Vorliegen einer CKD gescreent wird. Selbst bei einer laborchemisch nachgewiesenen CKD bleibt ein Großteil der Patienten ohne eine CKD-Diagnose nach ICD-10. Auch die empfohlene Erstlinientherapie mit einem RASi in Kombination mit einem SGLT-2i wird selten initiiert. Eine stärkere Sensibilisierung der Hausärzte für CKD und für die damit verbundenen gesundheitlichen Risiken ist daher erforderlich. Ein verstärkter interdisziplinärer Austausch könnte eine Verbesserung der Früherkennung und einen optimierten Einsatz gezielter CKD-Therapien unterstützen.

## Fazit für die Praxis

- Es besteht eine hohe Prävalenz der chronischen Nierenerkrankung („chronic kidney disease“ [CKD]) von 18,8 % bei Risikopatienten in Deutschland – ab einem Alter von 60 Jahren hat jeder vierte Risikopatient (24,7 %) eine CKD.
- Die Diagnosestellung bei vorliegender CKD ist gering: 83,5 % der Patienten mit CKD bleiben ohne Diagnose nach Internationaler statistischer Klassifikation der Krankheiten und verwandter Gesundheitsprobleme (ICD-10).
- Nur in 9,7 % der Fälle wird die CKD mit einem Renin-Angiotensin-System-Hemmer in Kombination mit einem Natrium-Glukose-Kotransporter-2-Hemmer behandelt – trotz des Potenzials zur Reduktion kardiorenaler Komplikationen und der Gesamtmortalität.
- Eine stärkere Sensibilisierung der Hausärzte für dieses unterschätzte Krankheitsbild ist erforderlich. Ein verstärkter interdisziplinärer Austausch könnte zur Verbesserung der Früherkennung und zum vermehrten Einsatz gezielter CKD-Therapien beitragen.
